# Risk factors associated with owner‐reported sleep disturbances in Nordic horses

**DOI:** 10.1111/evj.14560

**Published:** 2025-07-24

**Authors:** H. Suomala, I. Brotherus, L. Hänninen, E. Ternman, A. K. Mykkänen

**Affiliations:** ^1^ Department of Equine and Small Animal Medicine Faculty of Veterinary Medicine, University of Helsinki University of Helsinki Finland; ^2^ Equine Information Center Kiuruvesi Finland; ^3^ Department of Production Animal Medicine Faculty of Veterinary Medicine, University of Helsinki University of Helsinki Finland; ^4^ Faculty of Bioscience and Aquaculture Nord University Steinkjer Norway

**Keywords:** bedding, behaviour, horse, husbandry practices, sleep, welfare

## Abstract

**Background:**

Very little is known about sleep disturbances in horses, although several management or animal‐based factors may contribute to an increased risk of disturbances.

**Objectives:**

To investigate factors related to rest and sleep behaviour of horses kept in stalls as perceived by Nordic horse owners or caretakers and their association with suspected sleep disturbances (SSD).

**Study Design:**

Cross‐sectional survey.

**Methods:**

The 40 question online international survey included a convenience sample of horse owners or caretakers with horses over 4 years old kept in the same individual stalls for at least the last three previous months. Multivariate binary logistic regression was used to generate models of the factors associated with the SSD and their possible causes and consequences for the horses.

**Results:**

Overall, 5.0% of respondents (88/1749) suspected that their horse had sleep disturbances. The horse‐specific factors which were associated with SSD (adjusted OR; 95% CL) were age in years (1.12; 1.07–1.16), withers height (1.05; 1.03–1.08), current illness/injury (3.14; 1.92–5.15), if the horse was not seen laying down (27.82; 8.72–88.75), nightly injuries (9.16; 3.36–25.00), loss of balance/unexpected fall (40.30; 16.77–96.87), daytime drowsiness (3.85; 1.68–8.82) and difficulties lying down/getting up (16.33; 6.02–44.30). The environment‐specific factors associated with SSD were hard and dry (3.50; 1.56–7.84) and hard and wet (8.63; 2.41–30.94) lying surface.

**Main Limitations:**

Self‐selection enrolment of participants may limit the generalisability of the findings.

**Conclusions:**

Several environmental and horse‐related factors were found to be associated with SSD in horses, including hard and dry/wet lying surfaces.

## Resumo

**Contexto:**

Pouco se sabe sobre distúrbios do sono em cavalos, embora diversos fatores relacionados ao manejo ou ao próprio animal possam contribuir para um risco aumentado desses distúrbios.

**Objetivos:**

O objetivo deste estudo foi investigar os fatores relacionados ao comportamento de descanso e sono de cavalos mantidos em baias, e sua associação com suspeitos distúrbios de sono (SDS), de acordo com observações e relatos de proprietários ou cuidadores de cavalos nórdicos.

**Delineamento do estudo:**

Uma pesquisa internacional online, voltada para proprietários e cuidadores de cavalos, com quarenta perguntas foi realizada com base em uma amostragem por conveniência.

**Métodos:**

A pesquisa incluiu proprietários ou cuidadores de cavalos com idade acima de quatro anos, mantidos nas mesmas baias individuais por pelo menos os últimos três meses. Utilizou‐se regressão logística binária multivariada para gerar modelos dos fatores associados aos SDS, bem como suas possíveis causas e consequências para os cavalos.

**Resultados:**

No total, 5,0% dos respondentes (88/1749) suspeitavam que seu cavalo apresentava distúrbios do sono. Os fatores específicos do cavalo que se associaram aos SDS (OR ajustado; IC 95%) foram: idade em anos (1,12; 1,07 a 1,16), altura da cernelha (1,05; 1,03 a 1,08), doença ou lesão atual (3,14; 1,92 a 5,15), se o cavalo não era visto deitado (27,82; 8,72 a 88,75), lesões noturnas (9,16; 3,36 a 25,00), perda de equilíbrio/quedas inesperadas (40,30; 16,77 a 96,87), sonolência durante o dia (3,85; 1,68 a 8,82) e dificuldade para deitar/levantar (16,33; 6,02 a 44,30). Os fatores ambientais associados aos SDS incluíram superfície dura e seca (3,50; 1,56 a 7,84) e dura e molhada (8,63; 2,41 a 30,94).

**Principais limitações:**

A inscrição voluntária dos participantes pode limitar a generalização dos resultados.

**Conclusões:**

Vários fatores ambientais e relacionados aos cavalos foram associados ao SDS, incluindo superfícies duras e secas/molhadas para os cavalos se deitarem.

## 1 INTRODUCTION

Sleep disturbances and lack of adequate sleep are detrimental to the health and welfare of all mammals.^1^ Therefore, the characterisation of horse sleep and the factors that influence it is a growing area of interest.^2^, ^3^ Similar to many other species, sleep disturbances have also been recognised in domestic horses, and may even be relatively common.^4^, ^5^ However, neither intrinsic nor environmental factors associated with these disturbances have been extensively studied. Identifying the underlying causes of sleep disturbances in horses is important to ensure adequate sleep and to improve welfare.^3^

Horses are polyphasic sleepers with multiple sleep episodes across the 24 h period.^3^ The total daily sleep time for horses has been estimated to be 3–5 h.^2^ As in many other species, sleep episodes can be divided into stages: non‐rapid eye movement (NREM) and rapid eye movement (REM) sleep.^3^ NREM sleep takes place with the horse standing or in sternal recumbency. For REM sleep, muscle atonia requires that horses must lie down in either sternal or lateral recumbency.^4^, ^6^ Therefore, it is crucial that they are provided with the opportunity to do so to avoid sleep loss.

Many different factors can influence horses' willingness or opportunity to lay down and therefore interfere with the possibility for the horse to engage in sleep. Examples include the amount^6^, ^7^ and type of bedding material,^8^, ^9^, ^10^, ^11^, ^12^ hospitalisation and severity of disease,^13^ pain,^14^ stall size,^15^ feeding practices,^16^ amount of physical activity,^17^ and social rank in the herd,^18^, ^19^, ^20^ among others. While older literature often refers to sleep disturbances as narcolepsy, true familial narcolepsy is extremely rare in horses.^4^, ^21^, ^22^

Observing the horse's behaviour and related changes allows the identification of factors that may indicate sleep disturbances in horses. Equine sleep disturbances have been associated with excessive daytime sleepiness,^4^ as well as impaired cognitive performance.^6^, ^23^ Injury can also arise in horses that present with sleep deprivation, since REM‐sleep deficiency can manifest with atonic collapses.^5^ These collapses are often easily recognised as an indicator of sleep disturbance, but it is also likely that other forms of impaired sleep can substantially affect equine welfare.^3^

This study aimed to explore the factors influencing rest and sleep behaviour of horses housed in stalls, as perceived by horse owners or caretakers, and to examine their potential association with respondent‐reported sleep disturbances (SSD). Our study questions were: (1) Are certain types of horses more prone to/more at risk of SSD than others? (2) Are certain types of husbandry conditions associated with the SSD in horses? (3) What are the most typical behavioural traits of horses suspected of having SSD? Our hypothesis was that there are similarities between horses with SSD and their housing conditions, compared to horses without SSD.

## 2 MATERIALS AND METHODS

### 2.1 Data collection tools

The horse owners or caretakers, who were involved with the daily activities of the horse on a regular basis were requested to fill out an online questionnaire. The questionnaire was administered using online survey software (XM Qualtrics) with responses collected between December 2022 to April 2023 (Data S1). The questions were available in Finnish, Swedish, Norwegian and English and they were divided into four different topics: (1) Horse basic data (14 questions); (2) Stable environment (14 questions); (3) Social interaction and possibility for contact with other horses indoors (2 questions) and (4) Horse's rest behaviour (9 questions) (Table 1). The respondents were asked ‘Do you think the horse has problems related to rest or sleep?’ Inclusion criteria were that the horse had to be at least 4 years old and housed for at least 3 months in the same stable in a single stall.

**TABLE 1:** The summarised version of the questionnaire.

**(I) Horse basic data**
(1) Horse/Pony Breed
(2) Horse's age (years)
(3) Horse's height (estimate/measurement in cm)
(4) Sex
(5) Has the mare had foals?
(6) When did the mare have her last foal?
(7) Has the foal been weaned?
(8) When was the foal weaned?
(9) Which of the following is the horse currently mainly used for: Riding, Trotting, Gallop Racing, Breeding, Work (Forestry), Riding school teaching, Equitherapy, Other, please name
(10) Is the horse currently used for: Competing, Hobby/Leisure
(11) Does the horse currently have an illness or an injury?
(12) What illness/injury does the horse have?
(13) Is the horse currently on medication?
(14) Is the horse on pergolide (Prascend© or other)
**(II) Stable environment**
(1) Country of horse residence
(2) How many immediate neighbouring horses does the horse have in the stable?
(3) Does the horse have visual contact with other horses from her/his box?
(4) Stall size, width (cm)
(5) Stall size, length (cm)
(6) When does the stable close at night (last person leaves the stable)?
(7) When does the stable open in the morning (first person comes to the stable)?
(8) Is the horse fed in during the quiet hours (e.g., automatic feeder)?
(9) Does the horse use one stall in turns with another horse (e.g., one horse stays in the stall for the day and another for the night)?
(10) What bedding material is usually used?
(11) Is there a rubber mat on the floor of the stall?
(12) What is the amount of bedding material in the stall in the spot where the horse is lying (thickness in centimetres, preferably measure with a measurement tape)?
(13) Can you fall on your knees in the spot where the horse is lying down?
(14) Do your knees get wet (visible wet stains on your clothes) if you stay on your knees in the stall for 15 s?
**(III) Social interactions and possibility for contact with other horses indoors**
(1) Can the horse touch other horses from the pen?
(2) Does your horse usually interact with neighbouring horses outside feeding times?
**(IV) Horse's rest behaviour**
(1) I or somebody else have seen the horse lying down
(2) Where does the horse lie down?
(3) How often do you think the horse is lying down?
(4) I have seen the horse rolling in any location?
(5) Have you observed the following behaviours within the last year:
The horse has difficulties for lying down (e.g., stiffness or throwing the body down) Getting up is slow or difficult The horse has been leaning against the wall in the stall seemingly without possibility to move The horse has been seizing while recumbent (epilepsy‐like seizure) The horse is sleepy during the day I have not observed above mentioned behaviours I have observed some other abnormal behaviour, please describe
(6) The horse has been injured during the night within the last year?
(7) If the horse has been injured by night within the last year, the injuries have been located: Dorsal aspect of the front knees (carpi), Dorsal aspect of the fetlocks, Hocks, In the head, Elsewhere, where?
(8) The horse has lost its balance/fallen unexpectedly without a clear cause (e.g., in the stall, on the paddock, while being groomed) within the last year?
(9) Do you think the horse has problems related to rest or sleep?
(10) My observations that I have used for answering are based on: Video recordings, Live observations.

The respondents were advised to measure bedding depth in centimetres and assess bedding softness and wetness using the following test: dropping to the knees from a standing position onto the bedding (softness) and checking if the knees remained dry after kneeling for 15 s (wetness). The bedding was instructed to be spread out evenly before the testing. If dropping to the knees was not possible or caused pain, the sleeping surface was classified as hard, and if dropping to the knees was possible and did not cause pain, the sleeping surface was classified as soft. If the knees got wet within 15 s, the sleeping surface was classified as wet, and if they remained dry, it was classified as dry.^24^

### 2.2 Sample size and data analysis

The sufficient sample size was calculated to be 1050 horses using an estimated prevalence of SDD of 5% (proportion of soft bedding 0.4 in NSSD horses versus 0.2 in SDD horses, using power of 80% and significance of 5%) (https://epitools.ausvet.com.au).

If the response option ‘I don't know’ was used in any of the answers when investigating the relationship between SSD and the factors related to horses (the housing environment and behaviour), the question was excluded from the data.

For statistical analyses the knee test results (*n* = 1749) were categorised into four different comfort classes: SoftDry (*n* = 1474), SoftWet (*n* = 42), HardDry (*n* = 73) and HardWet (*n* = 14). No additional information was available for 146 cases. Different bedding materials (*n* = 1749) were further classified into the following six categories: Straw (*n* = 151), Pelleted (straw pellet, wood pellet, reed canary grass pellet, *n* = 236), Non pelleted (shavings, saw dust, peat, hemp, flax, *n* = 917), Straw combinations (*n* = 139), Peat combinations (*n* = 204) and Others (*n* = 102).

For the preliminary statistics, the association of all factors with suspected sleep disturbances (SSD) was analysed using Chi Square tests (Table 2), and differences in age, withers height (cm) and amount of bedding material (cm) between SSD (yes/no) were analysed with Mann–Whitney *U*‐tests (Tables S1 and S2).

**TABLE 2:** The results of the univariable analysis (chi‐square and Fisher's exact tests) of the behavioural factors associated with respondent‐reported suspected sleep disturbances in horses.

Variable	*N*	NSSD group	SSD group	*p*‐value	*X* ^2^
Observed rolling	1736	1650	86	<0.001	35.3
No	54	2.5% a (42/1650)	14.0% b (12/86)		
Yes	1682	97.5% a (1608/1650)	86.0% b (74/88)		
Observed lying down	1504	1450	54	<0.001	203.4
No	32	1.1% a (16/1450)	29.6% b (16/54)		
Yes	1472	98.9% a (1434/1450)	70.4% b (38/54)		
Observed lying frequency	1659	1595	64	<0.001	223.1
Daily	1522	93.5% a (1492/1595)	46.9% b (30/64)		
Min. once/week	115	5.8% a (92/1595)	35.9% b (23/64)		
Couple of times/month	15	0.4% a (7/1595)	12.5% b (8/64)		
Less often	7	0.3% a (4/1595)	4.7% b (3/64)		
Loss of balance/unexpected fall (min. once/year)	1749	1661	88	<0.001	391.3
No	1657	97.2% a (1614/1661)	48.9% b (43/88)		
Yes	92	2.8% a (47/1661)	51.1% b (45/88)		
Social interactions	1700	1616	84	<0.05	7.8
Kicking, biting or similar	78	4.3% a (69/1616)	10.7% b (9/84)		
Sniffing, grooming or similar	1226	72.5% a (1171/1616)	65.5% a (55/84)		
No interaction	396	23.3% a (376/1616)	23.8% a (20/84)		
Difficulties of lying down/getting up	1749	1661	88	<0.001	126.9
No	1672	96.9% a (1609/1661)	71.6% b (63/88)		
Yes	77	3.1% a (52/1661)	28.4% b (25/88)		
Daytime sleepiness	1749	1661	88	<0.001	136.3
No	1565	91.5% a (1519/1661)	52.3% b (46/88)		
Yes	184	8.5% a (142/1661)	47.7% b (42/88)		

*Note*: Summary of animal and animal‐based explanatory variables and their association with an outcome; no suspected sleep disturbances (NSSD) versus suspected sleep disturbances (SSD). Letters within rows denote significant differences between NSSD and SSD.

All factors were divided into possible causes and consequences of SSD, and initially two binary logistic regression models were created in which SSD was a dependent variable. The initial causal variables were horse breed, age, withers height, current use (competition/leisure horse), illness/injury, medication and bedding comfort class. The initial consequence variables were loss of balance/unexpected fall, nighttime injuries, location of nighttime injury, negative interaction with other horses, difficulty in lying down/standing up, rolling, lying down and its frequency, and perceived daytime sleepiness. The method of variable entry was the Enter method. Those variables that did not improve the model were removed before running the final models. Hosmer–Lemeshow goodness‐of‐fit statistic was used to assess how well the chosen model fits the data.

The final model based on horse and environmental risk factors included age in years, withers height (cm), current illness/injury, and HardDry and HardWet lying area. The final model based on cause and effect factors included losing balance/falling unexpectedly at least once per year, not seen to be lying down, being injured at night, difficulties lying down/getting up, and perceived daytime sleepiness.

Statistical analyses were conducted with IBM SPSS Statistics for Windows, version 28 (IBM Corp.).

## 3 RESULTS

In total 2578 responses were received, of which a total of 1749 responses were complete and met the inclusion criteria for the study. Most horses lived in Finland (56.6%), Sweden (31.6%) and Norway (10.7%). Overall, 1582 respondents answered the question on the current use of the horse, and 72.1% of those horses were leisure horses and 27.9% were competition horses. The most common horse breeds (*n* = 1731) were Coldblood (32.9%), Warmblood (26.0%) and Standardbred (23.2%). Among the horses, 45.7% were mares, 49.6% were geldings, and 4.6% were stallions. The horses were divided into two groups of equal sizes based on age: 4–12 years (50.3%) and 13–35 years (49.7%). In total, 5.0% of respondents (88/1749) reported SSD.

### 3.1 Horse‐related factors

A higher proportion of horses with SSD (*n* = 88) were older (age 13–35 years), taller (withers height >159 cm) and Warmblood (*p* < 0.001 for all) than horses without SSD. A higher proportion of horses with SSD were used for leisure purposes (*p* < 0.001) than horses without SSD. There was no observed sex difference related to SSD. However, a higher proportion of mares with SSD were primiparous or multiparous than mares without SSD (*p* < 0.05).

Current illness or injury and horses receiving medication were more common for those with SSD than without (*p* < 0.001 and *p* = 0.008, respectively). Horses with SSD were reported to be injured more often at night, and the injuries were on the dorsal aspect of the fetlock (*p* < 0.001 and *p* = 0.003, respectively) than horses without SSD. Detailed information on horse‐related factors with and without SSD is presented in Table S1.

### 3.2 Environmental‐related factors

Non‐pelleted bedding materials, including shavings, sawdust, peat, hemp, and flax were the most used (52.4%). Overall, 3.5% of respondents reported wet bedding in the knee test, and 5.4% reported hard bedding. Both wet and hard bedding as estimated in the knee test were more often associated with SSD (*p* < 0.001 for both) compared to those with no suspected sleep disturbances (NSSD). Other environmental‐related factors (type of bedding material, bedding thickness ≤5 cm or >5 cm, rubber‐mat on the floor, stall size, access to visual contact with other horses, access to muzzle contact with other horses, feeding horses in the quiet hours) were not associated with SSD. Detailed information on environmental‐related factors with and without SSD is presented in Table S2.

### 3.3 Behaviour‐related factors

In the initial analyses several behavioural traits were statistically associated with horses with SSD (*p* < 0.05 for all). A lower proportion of SSD horses rolled or lay down than horses with NSSD. In contrast, a higher proportion of SSD horses unexpectedly lost their balance, showed more aggressive behaviour towards other horses (e.g., kicking or biting), had difficulties lying down/getting up, and were sleepy during the day than NSSD horses (Table 2). The horses' behaviour was found to differ between SSD and NSSD in terms of lying behaviour and its frequency (Table 2).

### 3.4 Logistic model results

The final logistic model on factors of possible causes for SSD showed that hard and dry, as well as hard and wet beddings, as estimated by the knee test, were more often associated with SSD. There were statistically significant positive associations with increasing age, increasing withers height, and the presence of disease or injury with SSD (Table 3).

**TABLE 3:** Resultant risk factors associated with suspected sleep disturbances in individually housed horses from the multivariate binary logistic regression model.

Factor	*B*	S.E.	Adjusted OR (95% CI)	*p*‐value
Age (years)	0.109	0.02	1.12 (1.07–1.16)	<0.001
Withers height (cm)	0.052	0.01	1.05 (1.03–1.08)	<0.001
Illness/Injury	1.146	0.25	3.14 (1.92–5.15)	<0.001
Hard + dry lying area according to the knee test	1.253	0.41	3.50 (1.56–7.84)	0.002
Hard + wet lying area according to the knee test	2.156	0.65	8.63 (2.41–30.94)	<0.001
Constant	−13.524	1.94	0.00	<0.001

*Note*: OR for one‐unit increase in continuous variables. 0.245 Hosmer & Lemeshow.

Abbreviations: *B*, the estimated coefficient, with standard error, S.E.; Cl, confidence interval; OR, odds ratio.

A model of possible factors associated with SSD showed that loss of balance, not observed lying down, injury at night, difficulty getting up/down and daytime sleepiness were positively associated with SSD (Table 4).

**TABLE 4:** Possible factors associated with suspected sleep disturbances in individually housed horses from the multivariate binary logistic regression model.

Factor	*B*	S.E.	Adjusted OR (95% CI)	*p*‐value
Loss of balance/unexpected fall (min. once/year)	3.696	0.45	40.30 (16.77–96.87)	<0.001
Not observed lying down	3.326	0.59	27.82 (8.72–88.75)	<0.001
Nighttime injury	2.215	0.51	9.16 (3.36–25.00)	<0.001
Lying down/getting up difficulties	2.793	0.51	16.33 (6.02–44.30)	<0.001
Daytime sleepiness	1.348	0.42	3.85 (1.68–8.82)	<0.001
Constant	−5.449	0.36	0.004	<0.001

*Note*: OR for one‐unit increase in continuous variables. 0.124 Hosmer & Lemeshow.

Abbreviations: *B*, the estimated coefficient, with standard error, S.E; OR, odds ratio; Cl, confidence interval.

## 4 DISCUSSION

This study investigated potential risks to equine sleep disturbance. We found that taller size, older age, and keeping the horse on hard and wet bedding were more often associated with suspected sleep disturbances (SSD) compared to other horses. Abnormal resting behaviours such as difficulties in lying down and getting up, or not being seen lying at all, and the tendency to be sleepy during the day were associated with horse owners or caretakers suspecting sleeping disturbances in horses. In addition, horses that lost their balance unexpectedly or had nightly traumas were associated with SSD. This is, to our knowledge, the first published study on observed horse behaviours, health and environmental factors associated with suspected sleep disturbances in horses.

### 4.1 Horse‐specific factors associated with sleep disturbances

The horses with SSD were older and more likely to have a disease or injury than horses with no SSD (NSSD). Our results were somewhat similar to those reported by Oliveira et al. (2022) in which severe osteoarthritis reduced lying down and the duration thereof in hospitalised horses.^13^ Several painful medical conditions may affect a horse's willingness to lie down, such as multiple degenerative joint diseases, old fractures, gastrointestinal diseases or neurological diseases that cause discomfort when getting up and down.^4^ On the other hand, our results differ from those of Keleman,^25^ who found that neither age nor lameness was associated with altered lying time. However, it is possible that the old and tall horses in our study had underlying undiagnosed diseases such as osteoarthritis that made them less willing to lie down on a hard surface.^26^ Previously, shifting weight and unstable resting were found to be potential indicators for distinguishing horses with pain from those free of pain.^14^ However, in the current study, we did not attempt to recognise pain as a potential cause for SDD.

Sleep deprivation was suspected more often in leisure horses than in competition horses, similar to previous studies showing that exercised horses spent more time lying than non‐exercised horses.^17^ More research is needed, however, on the relationship between sleep and exercise in horses. Our analysis shows that horses with SSD were more often Warmblood than Coldblood. Although breed is associated with use, former racehorses can be used as hobby horses as they retire. The final model identified no significant association between breed and SSD. On the other hand, breed is associated with withers height and, in our study, the mean of NSSD withers height was 156.7 cm (SD 0.38) and SSD horses 163.5 cm (SD 1.40). The sex of the horse was not related to SSD, although sex has previously been shown to be associated with resting behaviour^27^: mares lay down twice as long as males and mares lay down mostly at night, accounting for 90% of the total daily lying time. In contrast, geldings and stallions lay down almost exclusively at night. We noticed in the initial analyses that foaled mares accounted for a higher proportion of horses suspected of sleep disturbance. This is supported by the finding that a few high‐risk pregnant mares hospitalised in their last month of pregnancy do not lie down at all and have multiple episodes of knuckling and almost collapsing.^4^ Future studies on this topic are therefore recommended.

### 4.2 Husbandry conditions associated with rest and suspected sleep disturbances

Although there were several possible sleep disturbance factors related to the horse stable environment in the questionnaire, only the uncomfortable resting surface was significantly associated with an increased risk for SSD. If the knee test result indicated hard and wet bedding, the SSD was 10 times more likely, and if the bedding was hard and dry, the suspected sleep disorder was 5 times more likely. This is in line with Greening et al.,^6^ who showed in an experimental setting that horses spent less time in lateral REM and sternal NREM, and more time in standing NREM when kept on a harder bedding compared to a softer bedding.

We were unable to show that a particular bedding material or rubber mat was associated with SSD. However, the confidence intervals were rather wide, probably reflecting the diverse bedding materials reported in this study. Nevertheless, several previous studies have shown that bedding material affects horses' sleep and resting behaviour, including time spent recumbent,^7^ time spent in a lying position,^8^ and number of lying occurrences.^9^ Horses are also willing to use rubber mats as resting surfaces if they are at least minimally covered with natural bedding materials such as shavings.^19^ We suggest that for a horse, the comfort of the resting surface is more important than the bedding material per se. Although stall size has been reported to affect sleep in horses,^15^ our findings did not reveal a statistical association between stall size and SSD, even when the size of the horse was taken into account.

### 4.3 Behavioural changes associated with suspected sleep disturbances

We show here that horses with SSD were less likely to lie down or to roll and had more difficulties in lying down and getting up.^3^ However, some of the horses that did roll over and lie down were also suspected to have SDD. We believe that in some of these cases, the respondents observing nightly video recordings of their horse's behaviour caused the suspicion of SDD. However, most horses were not under camera surveillance, which might have led to both inability to detect disturbances as well as overinterpretation of daily behaviours being caused by a sleep disturbance.

If a horse was seen to sway for no reason, it was 40 times more likely to be suspected of having SSD. Also, failure to lie down and nocturnal fetlock injuries were strongly associated with SSD. Our results are similar to those reported by Fuchs et al.,^20^ where horses with collapses spent less time in REM sleep, which requires recumbency. Although initial analyses showed that negative social interactions, such as kicking or biting a neighbouring stabled horse, were associated with horses with SSD, this was not confirmed in the final modelling. However, sleep deprivation has been found to cause aggressive behaviour^28^ and increased anxiety^29^ in other animal species, such as rats. Further research is needed to establish whether these behaviours can be caused by abnormal sleep patterns in horses.

This is the first study, to our knowledge, of horse owners and caretakers on possible factors that may influence sleep disturbance in horses, and further research is needed to better understand the subject. Since the survey only investigates respondent perceptions, some horses with SSD might have been overlooked, and further clinical sleep studies including polysomnography and video monitoring of horses are needed. Some of the preceding questions in the questionnaire may have inadvertently prompted respondents either to suspect or not to suspect SSD in their horse. On the other hand, owners or caretakers with previous suspicion or special interest in their horses' sleep might have been more prone to answer the questionnaire. We suggest that there are a multitude of factors associated with sleep disturbances in horses. The owner can improve the horse's sleeping environment by increasing its bedding comfort, which has previously been somewhat overlooked in equine management and is a potentially unattractive decision for the owner due to increasing costs.

## FUNDING INFORMATION

The study is part of the UNIHEPO project on equine sleep and rest, funded by the Ministry of Agriculture and Forestry, Finland.

## CONFLICT OF INTEREST STATEMENT

The authors declare no conflicts of interest.

## AUTHOR CONTRIBUTIONS

**Heli Suomala:** Conceptualization; data curation; investigation; validation; methodology; formal analysis; writing – original draft. **Iina Brotherus:** Writing – review and editing; conceptualization. **Laura Hänninen:** Conceptualization; methodology; validation; formal analysis; writing – original draft; investigation. **Emma Ternman:** Conceptualization; writing – review and editing; investigation; data curation. **Anna Kristina Mykkänen:** Conceptualization; supervision; funding acquisition; project administration; resources; writing – original draft; methodology; investigation.

## DATA INTEGRITY STATEMENT

H. Suomala had full access to all the data in the study and takes responsibility for the integrity of the data and the accuracy of the data analysis.

## ETHICAL ANIMAL RESEARCH

The Research Ethics Committee on Animal Research of the University of Helsinki has stated that the study is ethically acceptable (Statement no. 11/2023).

## INFORMED CONSENT

Completion of the questionnaire was taken as participant consent.

## Supporting information


**Data S1.** The complete questionnaire for horse owners/caretakers.

**Table S1.** The results of the univariable analysis (chi‐square test and Fisher's exact test) of the factors associated with respondent‐reported sleep disturbances in horses.

**Table S2.** The results of the univariable analysis (chi‐square test and Fisher's exact test) in factors associated with respondent‐reported sleep disturbances in horses.

## Data Availability

The data that support the findings of this study are openly available in https://zenodo.org at https://doi.org/10.5281/zenodo.15817991.
