# Supplementary material for: Risk factors associated with owner‐reported sleep disturbances in Nordic horses
Source: Equine Vet J. 2025 Jul 24;58(3):728–35. doi: 10.1111/evj.14560 (PMC13041605; doi:10.1111/evj.14560)
Supplement: Supplementary file 1 — Data S1. The complete questionnaire for horse owners/caretakers. [file EVJ-58-728-s003.pdf]

Data S1: Questionnaire in full.

This questionnaire is for those horse owners/horse caretakers whose horse lives in an individual box stall. The questionnaire is available in Finnish, Swedish, Norwegian, and English. Select the right language in the upper-right corner.

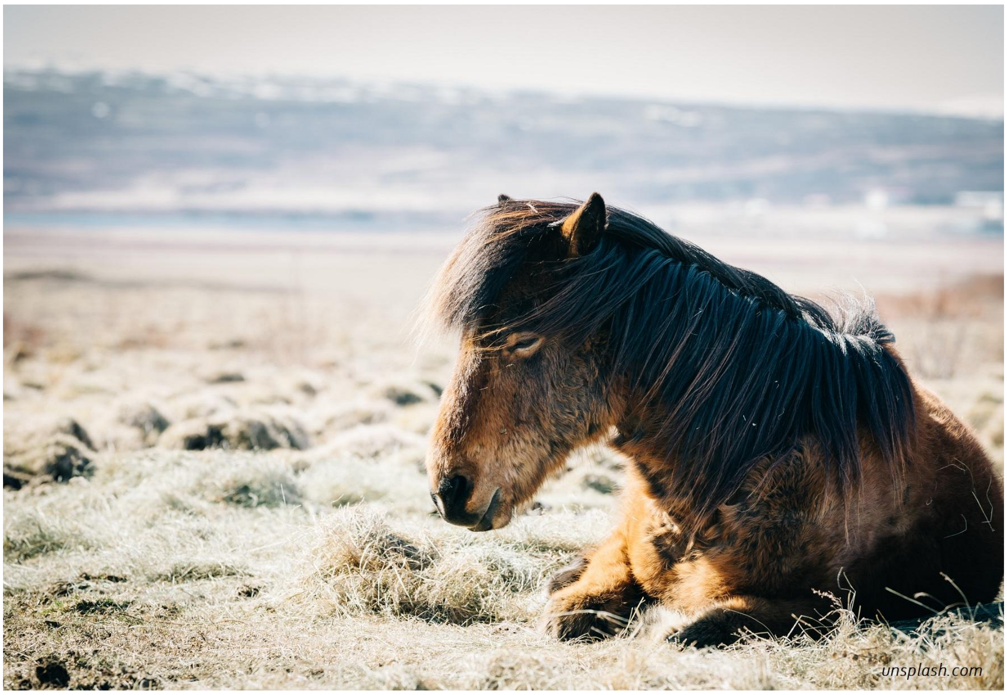

This questionnaire aims to study horse rest behavior and factors affecting it. The questionnaire should be filled by a person, that is involved with the daily activities of the horse on a regular basis. The horse should be over 4 years of age and have lived at least 3 months in the same individual box stall. It takes 10–15 minutes to fulfil the questionnaire. Please note that some of the questions are best answered if you are at the stable and have a measuring tape with you.

Two gift-cards worth 100 euros will be drawn among the respondents. If you wish to take part in the lottery, please click the link at the end of the questionnaire. The contact details for the lottery are not connected to the answers given.

1. HORSE BASIC DATA

Horse/Pony Breed:

[ ] Finnhorse

[ ] Swedish Coldblood Trotter

[ ] Norwegian Coldblood Trotter

[ ] Norwegian Fjord

[ ] Estonian horse

[ ] Standardbred

[ ] Warmblood

[ ] Arabian horse

[ ] Thoroughbred

[ ] Icelandic horse

[ ] Shetland pony

[ ] Gotland Russ

[ ] New Forest Pony

[ ] Connemara Pony

[ ] Other, please name

Horse’s age (years):

Horse’s height (estimate/measurement in centimeters):

Sex:

[ ] Stallion

[ ] Gelding

[ ] Mare

Has the mare had foals:

[ ] Yes

[ ] No

[ ] I don´t know

When did the mare have her last foal? Month and year (For example, May 2022):

Has the foal been weaned:

[ ] Yes

[ ] No

When was the foal weaned? Month and year (For example, December 2022):

Which of the following is the horse currently mainly used for:

[ ] Riding

[ ] Trotting

[ ] Gallop Racing

[ ] Breeding

[ ] Work (Forestry)

[ ] Riding school teaching

[ ] Equitherapy

[ ] Other, please name:

Is the horse currently used for:

[ ] Competing

[ ] Hobby/Leisure

Does the horse currently have an illness or an injury?

[ ] No

[ ] Yes

[ ] I don´t know

What illness/injury does the horse have?

[ ] The horse has PPID (Cushing)

[ ] The horse has PPID (Cushing), but she/he also has other illness/injury. What other illness/injury does the horse have (please, name)?

[ ] The horse has only other illness/injury than PPID. What other illness/injury does the horse have (please, name)?

[ ] I don´t know

Is the horse currently on medication?

[ ] No

[ ] Yes

[ ] I don´t know

Is the horse on pergolide (Prascend© or other):

[ ] Yes

[ ] Yes, but she/he also has other medication (please, name)?

[ ] No. What other medication does the horse have (please, name)?

[ ] I don't know

2. STABLE ENVIRONMENT

Country of horse residence:

[ ] Finland

[ ] Sweden

[ ] Norway

[ ] Denmark

[ ] Germany

[ ] Poland

[ ] Estonia

[ ] Latvia

[ ] Lithuania

Other, please name

How many immediate neighboring horses does the horse have in the stable?

[ ] 0

[ ] 1

[ ] 2

[ ] 3

[ ] I don’t know

Does the horse have visual contact with other horses from her/his box?

[ ] Yes

[ ] No

[ ] I don’t know

Stall size, width (centimeters):

Stall size, length (centimeters):

When does the stable close at night (last person leaves the stable)?

[ ] 5 pm

[ ] 6 pm

[ ] 7 pm

[ ] 8 pm

[ ] 9 pm

[ ] 10 pm

[ ] The stable does not close

[ ] I don’t know

When does the stable open in the morning (first person comes to the stable)?

[ ] 5 am

[ ] 6 am

[ ] 7 am

[ ] 8 am

[ ] 9 am

[ ] I don’t know

Is the horse fed in the during the quiet hours (e.g., automatic feeder)?

[ ] Yes

[ ] No

[ ] I don’t know

Does the horse use one stall in turns with another horse (e.g., one horse stays in the stall for the day and another for the night)?

[ ] No

[ ] Yes, the horse stays the nights mostly outside in the paddock/pasture

[ ] Yes, the horse stays the nights mostly inside in the stall

[ ] I don’t know

What bedding material is usually used (you can pick many)?

[ ] Straw

[ ] Straw pellet

[ ] Peat

[ ] Wood pellet

[ ] Reed canary grass

[ ] Shavings

[ ] Saw dust

[ ] Hemp

[ ] Flax

[ ] No bedding is used

[ ] Other please name

[ ] I don’t know

Is there a rubber mat on the floor of the stall?

[ ] No

[ ] Yes

[ ] I don’t know

Please make sure that there is an even layer of bedding material in the stall (not a pile of freshly added bedding material), and answer the following questions:

What is the amount of bedding material in the stall in the spot where the horse is lying (thickness in centimeters, preferably measure with a measurement tape)?

Thickness (cm):

[ ] No bedding is used

Can you fall on your knees in the spot where the horse is lying down?

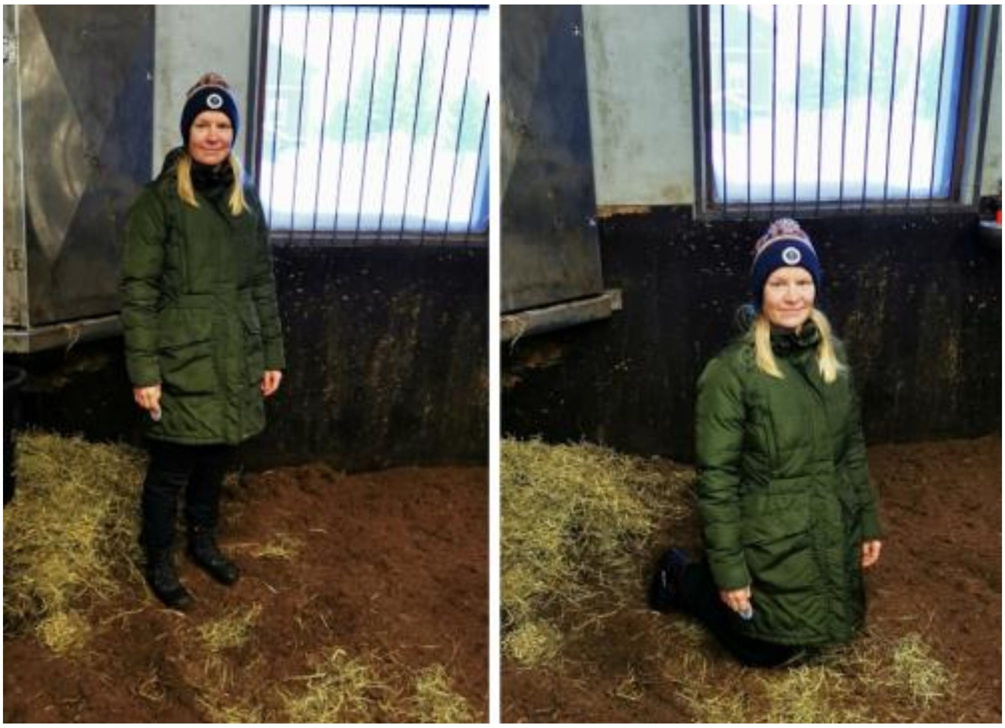

[ ] No, because it hurts

[ ] Yes, because it does not hurt

[ ] I don't know

Do your knees get wet (visible wet stains on your clothes) if you stay on your knees in the stall for 15 seconds?

[ ] No

[ ] Yes

[ ] I don’t know

3. SOCIAL INTERACTIONS AND POSSIBILITY FOR CONTACT WITH OTHER HORSES INDOORS

Can the horse touch other horses from the pen?

[ ] No

[ ] Yes

[ ] I don’t know

Does your horse usually interact with neighboring horses outside feeding times?

[ ] Kicking, biting or similar behaviors

[ ] Sniffing, grooming or similar behaviors

[ ] No interaction

[ ] I don’t know

4. HORSE’S REST BEHAVIOUR

I or somebody else have seen the horse lying down

[ ] Yes

[ ] No

[ ] I don’t know

[ ] No, but I assume it has been laying, because there has been bedding material or dirt on the horse

Where does the horse lie down (you can pick many)?

[ ] In the stall

[ ] In the paddock

[ ] On pasture

[ ] Somewhere else, where?

How often do you think the horse is lying down?

[ ] Daily

[ ] At least once a week

[ ] A couple of times per month

[ ] Less often

[ ] I don’t know

I have seen the horse rolling in any location?

[ ] Yes

[ ] No

[ ] I don´t know

Have you observed the following behaviors within the last year (you can pick many):

[ ] The horse has difficulties for lying down (e.g. stiffness or throwing the body down)

[ ] Getting up is slow or difficult

[ ] The horse has been leaning against the wall in the stall seemingly without possibility to move

[ ] The horse has been seizuring while recumbent (epilepsy-like seizure)

[ ] The horse is sleepy during the day

[ ] I have not observed above mentioned behaviors

[ ] I have observed some other abnormal behaviour, please describe:

The horse has been injured during the night within the last year?

[ ] Yes

[ ] No

[ ] I don´t know

If the horse has been injured by night within the last year, the injuries have been located:

[ ] Dorsal aspect of the front knees (carpi)

[ ] Dorsal aspect of the fetlocks

[ ] Hocks

[ ] In the head

[ ] Elsewhere, where?

The horse has lost its balance/fallen unexpectedly without a clear cause (e.g. in the stall, on the paddock, while being groomed) within the last year?

[[No

[ ] Yes, once

[ ] Yes, daily

[ ] Yes, weekly

[ ] Yes, Monthly

[ ] I don´t know

Do you think the horse has problems related to rest or sleep?

[ ] No

[ ] Yes. What do you think is causing the problem?

[ ] I don´t know

My observations that I have used for answering are based on (you can choose both):

[ ] Video recordings

[ ] Live observations
