# Supplementary material for: Risk factors associated with owner‐reported sleep disturbances in Nordic horses
Source: Equine Vet J. 2025 Jul 24;58(3):728–35. doi: 10.1111/evj.14560 (PMC13041605; doi:10.1111/evj.14560)
Supplement: Supplementary file 2 — Table S1. The results of the univariable analysis (chi‐square test and Fisher's exact test) of the factors associated with respondent‐reported sleep disturbances in horses. [file EVJ-58-728-s002.docx]

**Table S1:** The results of the univariable analysis (Chi-Square Test and Fisher's Exact Test) of the factors associated with respondent-reported sleep disturbances in horses.

Summary of animal and animal-based explanatory variables and their association with an outcome; no suspected sleep disturbances (NSSD) vs. suspected sleep disturbances (SSD).

| **Variable** | **n** | **NSSD group** | **SSD group** | **P-value** | **X^2^** |
| --- | --- | --- | --- | --- | --- |
| **Breed** | 1731 | 1643 | 88 | <0.001 | 26.6 |
| Coldblood | 570 | 33.7% a (554/1643) | 18.2% b (16/88) |  |  |
| Standardbred | 401 | 23.5% a (386/1643) | 17.0% a (15/88) |  |  |
| Warmblood | 450 | 24.8% a (408/1643) | 47.7% b (42/88) |  |  |
| Thoroughbred | 43 | 2.4% a (39/1643) | 4.5% a (4/88) |  |  |
| Pony | 218 | 12.7% a (209/1643) | 10.2% a (9/88) |  |  |
| Other | 49 | 2.9% a (47/1643) | 2.3% a (2/88) |  |  |
| **Gender** | 1749 | 1661 | 88 | >0.05 | 2.7 |
| Mare | 800 | 45.8% a (760/1661) | 45.5% a (40/88) |  |  |
| Gelding | 868 | 49.4% a (821/1661) | 53.4% a (47/88) |  |  |
| Stallion | 81 | 4.8% a (80/1661) | 1.1% a (1/88) |  |  |
| **Age (years)** | 1749 | 1661 | 88 | <0.001 | 23.6 |
| 4–12 | 879 | 51.6% a (857/1661) | 25.0% b (22/88) |  |  |
| 13–35 | 870 | 48.4% a (804/1661) | 75.0% b (66/88) |  |  |
| **Withers height (cm)** | 1722 | 1634 | 88 | <0.001 | 13.9 |
| ⋜ 159 | 802 | 47.6% a (778/1634) | 27.3% b (24/88) |  |  |
| > 159 | 920 | 52.4% a (856/1634) | 72.7% b (64/88) |  |  |
| **Current use** | 1582 | 1504 | 78 | <0.001 | 16.6 |
| Competing | 441 | 28.9% a (435/1504) | 7.7% b (6/78) |  |  |
| Hobby/Leisure | 1141 | 71.1% a (1069/1504) | 92.3% b (72/78) |  |  |
| **Illness/injury** | 1724 | 1646 | 78 | <0.001 | 41.1 |
| No | 1445 | 85.1% a (1400/1646) | 57.7% b (45/78) |  |  |
| Yes | 279 | 14.9% a (246/1646) | 42.3% b (33/78) |  |  |
| **Medication** | 1744 | 1656 | 88 | <0.05 | 8.5 |
| No | 1647 | 94.8% a (1570/1656) | 87.5% b (77/88) |  |  |
| Yes | 97 | 5.2% a (86/1656) | 12.5% b (11/88) |  |  |
| **Injured at night** | 1727 | 1643 | 84 | <0.001 | 167.9 |
| No | 1643 | 96.7% a (1588/1643) | 65.5% b (55/84) |  |  |
| Yes | 84 | 3.3% a (55/1643) | 34.5% b (29/84) |  |  |
| **Location of nightly injuries** | 80 | 51 | 29 | <0.05 | 15.9 |
| Front knees | 6 | 3.9% a (2/51) | 13.8% a (4/29) |  |  |
| Fetlocks | 16 | 9.8% a (5/51) | 37.9% b (11/29) |  |  |
| Hocks | 18 | 21.6% a (11/51) | 24.1% a (7/29) |  |  |
| Head | 12 | 19.6% a (10/51) | 6.9% a (2/29) |  |  |
| Other | 28 | 45.1% a (23/51) | 17.2% b (5/29) |  |  |
| **The mare has foaled** | 778 | 741 | 37 | <0.05 | 7.6 |
| No | 482 | 63.0% a (467/741) | 40.5% b (15/37) |  |  |
| Yes | 296 | 37.0% a (274/741) | 59.5% b (22/37) |  |  |
| **The foal is weaned** | 292 | 270 | 22 | >0.05 | 0.6 |
| No | 26 | 9.3% a (25/270) | 4.5% a (1/22) |  |  |
| Yes | 266 | 90.7% a (245/270) | 95.5% a (21/22) |  |  |

Note: Letters within rows denote significant differences between NSSD and SSD.
