# Supplementary material for: Risk factors associated with owner‐reported sleep disturbances in Nordic horses
Source: Equine Vet J. 2025 Jul 24;58(3):728–35. doi: 10.1111/evj.14560 (PMC13041605; doi:10.1111/evj.14560)
Supplement: Supplementary file 3 — Table S2. The results of the univariable analysis (chi‐square test and Fisher's exact test) in factors associated with respondent‐reported sleep disturbances in horses. [file EVJ-58-728-s001.docx]

**Table S2:** The results of the univariable analysis (Chi-Square Test and Fisher's Exact Test) of the factors associated with respondent-reported sleep disturbances in horses.

Summary of animal and husbandry/environment-based explanatory variables and their association with an outcome; no suspected sleep disturbances (NSSD) vs. suspected sleep disturbances (SSD).

| **Variable** | **n** | **NSSD group** | **SSD group** | **P-value** | **X^2^** |
| --- | --- | --- | --- | --- | --- |
| **Bedding material** | 1749 | 1661 | 88 | >0.05 | 4.9 |
| Straw | 151 | 8.9% a (147/1661) | 4.5% a (4/88) |  |  |
| Non-pelleted † | 917 | 52.7% a (876/1661) | 46.6% a (41/88) |  |  |
| Pelleted ‡ | 236 | 13.3% a (221/1661) | 17.0% a (15/1661) |  |  |
| Peat + other | 204 | 11.5% a (191/1661) | 14.8% a (13/88) |  |  |
| Straw + other | 139 | 7.9% a (131/1661) | 9.1% a (8/88) |  |  |
| Other combination | 102 | 5.7% a (95/1661) | 8.0% a (7/88) |  |  |
| **Bedding thickness (cm)** | 1749 | 1661 | 88 | >0.05 | 0.9 |
| ≤ 5 | 116 | 6.5% a (108/1661) | 9.1% a (8/88) |  |  |
| > 5 | 1633 | 93.5% a (1553/1661) | 90.9% a (80/88) |  |  |
| **Knee test(falling on knees is possible)** | 1628 | 1546 | 82 | <0.001 | 18.4 |
| No | 88 | 4.9% a (75/1546) | 15.9% b (13/82) |  |  |
| Yes | 1540 | 95.1% a (1471/1546) | 84.1% b (69/82) |  |  |
| **Knee test (knees get wet)** | 1698 | 1614 | 84 | <0.001 | 18.7 |
| No | 1639 | 97.0% a (1565/1614) | 88.1% b (74/88) |  |  |
| Yes | 59 | 3.0% a (49/1614) | 11.9% b (10/88) |  |  |
| **Rubber mat on the floor** | 1749 | 1661 | 88 | >0.05 | 2.2 |
| No | 1425 | 81.2% a (1348/1661) | 87.5% a (77/88) |  |  |
| Yes | 324 | 18.8% a (313/1661) | 12.5% a (11/88) |  |  |
| **Stall size (m²)** | 1632 | 1548 | 84 | >0.05 | 0.0 |
| ≤ 9 | 167 | 10.2% a (158/1548) | 10.7% a (9/84) |  |  |
| > 9 | 1465 | 89.8% a (1390/1548) | 89.3% a (75/84) |  |  |
| **Access to visual contact** | 1749 | 1661 | 88 |  | 1.9 |
| No | 36 | 2.2% a (36/1661) | 0.0% a (0/88) |  |  |
| Yes | 1713 | 97.8% a (1625/1661) | 100.0% a (88/88) |  |  |
| **Access to muzzle contact** | 1746 | 1658 | 88 | >0,05 | 0.5 |
| No | 558 | 31.8% a (527/1658) | 35.2% a (31/88) |  |  |
| Yes | 1188 | 68.2% a (1131/1658) | 64.8% a (57/88) |  |  |
| **Fed in the quiet hours**  **(e.g. automatic feeder)** | 1747 | 1659 | 88 | >0.05 | 0.1 |
| No | 1533 | 87.7% a (1455/1659) | 88.6% a (78/88) |  |  |
| Yes | 214 | 12.3% a (204/1659) | 11.4% a (10/88) |  |  |

Note: Letters within rows denote significant differences between NSSD and SSD. † Non-Pelleted= Shavings, saw dust, peat, hemp, flax, ‡ Pelleted=Straw pellet, wood pellet, reed canary grass pellet.
